# The Effects of the Toxic Cyanobacterium *Limnothrix* (Strain AC0243) on *Bufo marinus* Larvae

**DOI:** 10.3390/toxins6031021

**Published:** 2014-03-06

**Authors:** Olivia Daniels, Larelle Fabbro, Sandrine Makiela

**Affiliations:** School of Medical and Applied Sciences, Central Queensland University, Rockhampton 4701, Australia; E-Mails: l.fabbro@cqu.edu.au (L.F.); s.makiela@cqu.edu.au (S.M.)

**Keywords:** *Limnothrix*, *Geitlerinema*, toxic, cyanobacteria, histology, *Bufo marinus*, notochord, brain, liver, pancreas, BMAA

## Abstract

*Limnothrix* (strain AC0243) is a cyanobacterium, which has only recently been identified as toxin producing. Under laboratory conditions, *Bufo marinus* larvae were exposed to 100,000 cells mL^−1^ of *Limnothrix* (strain AC0243) live cultures for seven days. Histological examinations were conducted *post mortem* and revealed damage to the notochord, eyes, brain, liver, kidney, pancreas, gastrointestinal tract, and heart. The histopathological results highlight the toxicological impact of this strain, particularly during developmental stages. Toxicological similarities to β-*N*-Methylamino-l-alanine are discussed.

## 1. Introduction

*Limnothrix* (strain AC0243) specific research is in its infancy due to its toxicity only recently being discovered.The strain shares intrinsic morphological characteristics with *Geitlerinema* species with field samples often identified as *G. unigranulatum* (R.N.Singh) J.Komárek and M.T.P.Azevedo or *G. amphibium* (C.Agardh ex Gomont) Anagnostidis. However, isolates of this strain have been genetically matched to *Limnothrix redekei* (Van Goor) Meffert with a 99% similarity, using 16*s* *r*RNA gene sequencing analysis [1].

*Limnothrix* (strain AC0243) has been shown to produce a water-soluble cytotoxin(s) that inhibits protein synthesis while independently reducing adenosine triphosphate (ATP) concentrations [1,2]. The protein synthesis inhibition displayed by this strain showed similarities to cylindrospermopsin (CYN). However, high performance liquid chromatography photo diode array (HPLC-PDA) and liquid chromatography mass spectrometry (LC-MS) failed to detect CYN and deoxy-CYN associated with this material [1]. Extracts of the strain administered to mice via intraperitoneal (IP) injections resulted in heightened sensitivity and hind limb weakness, suggesting the possibility of neurotoxin exposure [2].

The most common cyanobacterial neurotoxins are anatoxin-a, anatoxin-a(s), and its analogues homoanatoxin-a, saxitoxin, and β-*N-*Methylamino-l-alanine (BMAA) [3,4,5,6,7]. Of these, the neurotoxic amino acid BMAA has been detected in many cyanobacterial species [5,8,9,10] and has been implicated in neurological diseases, such as motor neuron disease, amyotrophic lateral sclerosis/Parkinson’s dementia complex, and Alzeimer’s disease [8,11]. BMAA is a mixed receptor agonist, which impacts energy and amino acid metabolism in neonates [8,10,12,13,14,15,16,17]. Of note is the similarity in modification of energy metabolism produced as a result of exposure of a Vero cell line to *Limnothrix* (AC0243) and that produced in neonatal rats following exposure to BMAA, particularly with respect to the decrease in production of ATP [2,13].

The impacts of toxic cyanobacteria have been well documented in terms of fish and mammalian species [18,19,20,21,22,23,24,25,26]. However, the toxigenic effects on anurans are seldom reported. Elements of the anuran lifestyle are highly reliant upon water as a support medium, source of food, and source of water for hydration. Although mature anurans generally adapt a terrestrial lifestyle, they rely on water bodies for cutaneous water absorption as their only means for hydration [27,28,29,30,31]. Cutaneous water absorption may provide a mode of exposure to cyanotoxins, promoting morbidities or mortalities in exposed animals [32,33]. The larval stage of most species is spent entirely in the water often grazing on plant matter and cyanobacteria [34,35,36]. Larvae, also absorbing water cutaneously, present ideal subjects for examining the effects of normal routes of exposure to *Limnothrix* (strain AC0243) that may occur in their natural habitat. 

The benefit of using anuran larvae as test specimens introduces the opportunity to examine the toxigenic effects of this strain on development stages, particularly stages where at notochord is present. The notochord is critical for the formation of muscle tissue and skeletal development as well as acting as a signaling source for the development of other tissues [37,38,39,40,41]. Anuran notochord damage resulting from cyanotoxin exposure has not been well represented in current literature although development abnormalities have been noted [42,43]. Such exposure requires further investigation as toxic blooms are rarely considered in terms of further impacting anuran communities and populations. 

### 1.1. Aim of the Experiments

This study examines the effects of whole cells (live cultures) of toxin producing *Limnothrix* (strain AC0243) (100,000 cells mL^−1^) on anuran larvae, using *Bufo marinus* as a representative species. This research was aimed at further understanding the potential impact of exposure on an early developmental, stage as well as the environmental consequences of *Limnothrix* (strain AC0243) blooms. 

### 1.2. Animal Ethics Committee Approval

Central Queensland University Rockhampton Animal Ethics Committee (AEC) approval (approval number A10/05-259) was obtained prior to the commencement of the experiments. All experiments were conducted in accordance with the Australian Code of Practice for the Care and Use of Animals for Scientific Purposes [44].

## 2. Results and Discussion

### 2.1. Water Quality Testing

Results for water quality variables in both the control and treatment groups were similar for dissolved oxygen, water temperature, and ammonia. No significant differences in dissolved oxygen (% saturation) were established between the controls and the treatment group (Tamhene’s *Post Hoc* test (*p* = 0.556)). Similarly, water temperatures did not significantly vary between treatments (*p* = 0.994). The ammonia concentrations remained stable (<2 ppm) for both groups.

In contrast, the water quality results indicated that pH varied significantly between the controls and the treatment (100,000 cells mL^−1^ *Limnothrix* (strain AC0243) live cultures) (Tamhene’s *Post Hoc* test (*p* < 0.05)). Treatment flasks showed higher pH concentrations than the control flasks (Table 1). Previous research has shown that growth of cyanobacteria in high concentrations may cause an increase in the pH [45]. Drops in pH (pH < 6) have been shown to cause mortalities in anuran larvae either directly, by having a toxic effect on biota or indirectly, by causing an increase in toxicity of other chemicals or metals [46,47]. The guidelines for ‘normal’ pH range for tropical areas of Australia, including Rockhampton are between pH 6.00 and 8.00, based on freshwater lakes and reservoirs in the region [47]. Waters with pH 8 have not been shown to cause significant mortality risks to anuran larvae [48] and breeding ponds of *B. marinus* are generally alkaline [49], suggesting that the pH in these *B. marinus* experiments fell within a ‘normal’ range and had no direct effect on the animals. However, it is unclear if the increased pH had any impact on the toxicity of *Limnothrix* (strain AC0243) and can not be ruled out as an influencing factor.

### 2.2. Histology

Mortalities occurred in treatment animals (one on day one, and one on day three) resulting in the planned fourteen-day experiment being reduced to seven days, with the surviving animals then euthanized and processed for histology. No deaths occurred in the control group during this period. Cellular damage was identified in the brain, liver, kidneys, pancreas, gastrointestinal tract, heart, lung bud, eye, and gill tissues, as a result of exposure to *Limnothrix* (strain AC0243) live cultures (Table 2).

**Table 1:** Water quality means and standard deviations for controls and treatment flasks containing 100,000 cells mL^−1^ of *Limnothrix* (strain AC0243) live cultures.

Treatment	Variable	Mean	Std. deviation
Control	Dissolved oxygen (% saturation)	33.23	8.27
	pH	7.51	0.22
	Water temperature (°C)	19.75	0.77
	Ammonia concentration (ppm)	<2	N/A
100,000 cells mL^−1^	Dissolved oxygen (% saturation)	35.39	6.66
	pH	7.89	0.27
	Water temperature (°C)	19.98	0.67
	Ammonia concentration (ppm)	<2	N/A

**Table 2:** Histopathologies of *Bufo marinus* larvae exposed to 100,000 cells mL^−1^ of *Limnothrix* (strain AC0243) live cultures.

Body region	Pyknotic cells	Eosinophilic cells	Necrotic cells	Karyorrhexis	Inflammatory cells	Tissue vacuolation
Eyes					+	
Brain	+	+	+	+	+	+
Ventricle						+
Gill filaments			+		+	
Kidneys						+
Lung bud			+			
Liver		+			+	+
Pancreas	+	+	+			
Gastrointestinal tract			+			
Notochord						

**Figure 1:**
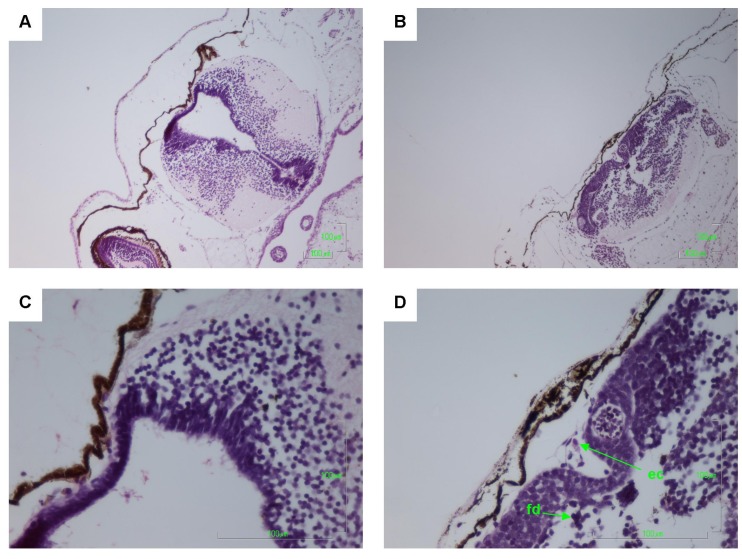
Haematoxylin and Eosin stained brain sections of *Bufo marinus* larvae exposed to 100,000 cells mL^−1^ of *Limnothrix* (strain AC0243) live cultures. (**A**) Control: Brain region at 100× magnification; (**B**) Test animal: Brain region at 100× magnification; (**C**) Control: Brain region at 400× magnification; (**D**) Test animal: Brain region at 400× magnification. Arrow indicates (ec) eosinophilic cells; (fd) fragmented grey matter infiltrating the diocoel.

**Figure 2:**
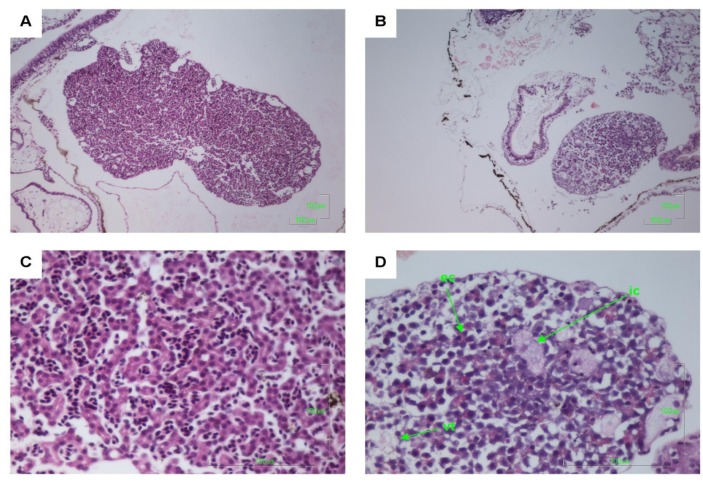
Haematoxylin and Eosin stained liver sections of *Bufo marinus* larvae exposed to 100,000 cells mL^−1^ of *Limnothrix* (strain AC0243) live cultures. (**A**) Control: Liver at 100× magnification; (**B**) Test animal: Liver at 100× magnification; (**C**) Control: Liver at 400×; (**D**) Test animal: Liver at 400 magnification. Arrows indicate (ic) unidentified cell type; (ec) eosinophilic cells; (vt) vacuolated tissue.

**Figure 3:**
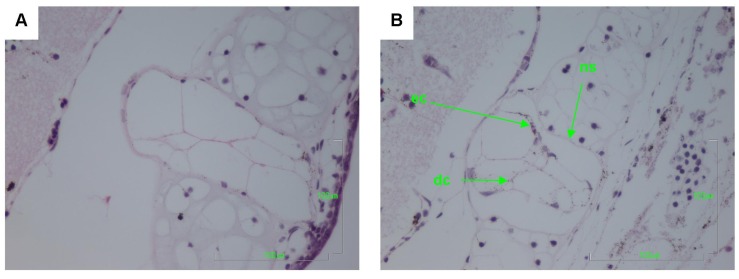
Haematoxylin and Eosin stained notochord sections of *Bufo marinus* larvae exposed to 100,000 cells mL^−1^ of *Limnothrix* (strain AC0243) live cultures. (**A**) Control: Notochord at 400× magnification; (**B**) Test animal: Notochord at 400× magnification. Arrow heads indicate; (ec) eosinophilic cells (ns) separation of the notochord from the chorda sheath; (dc) debris between chorda cells.

**Figure 4:**
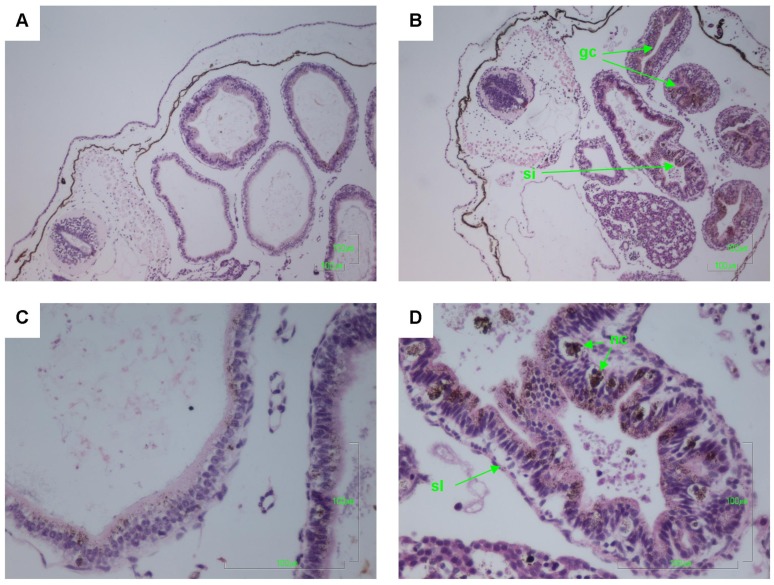
Haematoxylin and Eosin stained gastrointestinal tract (GI) sections of *Bufo marinus* larvae exposed to 100,000 cells mL^−1^ of *Limnothrix* (strain AC0243) live cultures. (**A**) Control: Gastrointestinal tract at 100× magnification; (**B**) Test animal: Gastrointestinal tract at 100× magnification; (**C**) Control: Gastrointestinal tract at 400× magnification; (**D**) Test animal: Gastrointestinal tract at 400× magnification. Arrows indicate (gc) narrowing to total closure of the GI tract; (si) increase in surface area of the epithelium; (nc) necrotic cells; (sl) separation of the outer longitudinal layer from the mucosa.

**Figure 5:**
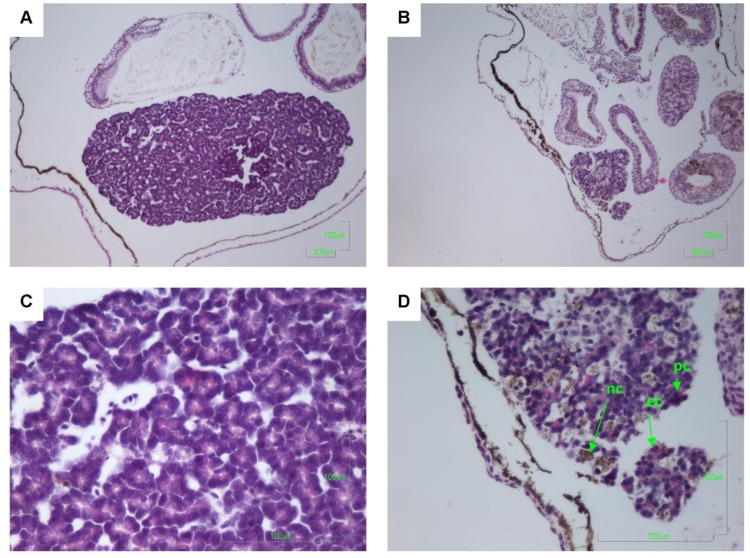
Haematoxylin and Eosin stained pancreas sections of *Bufo marinus* larvae exposed to 100,000 cells mL^−1^ of *Limnothrix* (strain AC0243) live cultures. (**A**) Control: Pancreas at 100× magnification. (**B**) Test animal: Pancreas at 100× magnification. (**C**) Control: Pancreas at 400× magnification. (**D**) Test animal: Pancreas at 400× magnification. Arrows indicate (nc) necrotic cells; (ec) eosinophilic cells; (pc) pyknotic cells.

Other pathologies were also seen in the *B. marinus* sections with the liver, pancreas, brain, notochord and gastrointestinal tract exhibiting extensive damage (Figure 1, Figure 2, Figure 3, Figure 4 and Figure 5). These other pathologies included unidentified cell types and deterioration of hepato-pancreatic region. These unidentified cell types were also seen on the lens epithelium of the eye. Brain sections showed an increased thickness of the diencephalon as well as fragmentation and infiltration of cellular debris into the diocoel. The notochord was affected with separation of the notochord from the chorda sheath and increased debris between chorda cells shown. The gastrointestinal tract was extensively affected, illustrated by separation of the outer longitudinal layer from the mucosa in the gastrointestinal tract, significant increase in surface area of the epithelium and narrowing to total closure of the gastrointestinal tract. 

Other, less extensive pathologies occurred in the heart, kidneys and gill filaments. Heart damage included vacuolated ventricular connective tissue that appeared more fibrous. The kidney tubules also appeared more fibrous. The gills showed smoothing (reduction in surface area) of the gill filaments.

Systemic toxicosis of exposed tadpoles suggests that the toxin(s) produced by *Limnothrix* (strain AC0243) is readily absorbed. Results of the mouse trials concluded damage to the kidneys, liver, gastrointestinal tract and lungs of treated animals [2]. Similar findings were recorded in these *B. marinus* larvae trials, with the addition of newly described injuries to the brain, notochord, and pancreas. Although mice exposed to cell extracts of the strain reportedly showed indications of possible neuropathy, the authors concluded that the neuropathy was likely a result of pain ensuing systemic toxicosis due to the atypical physiological symptoms exhibited by the mice and the lack of histological evidence of brain injury [2]. However, the lack of histological pathologies is reportedly typical for neurotoxin exposure [4,50]. Based on the current findings using a developmental stage rather than an adult stage, the possibility of *Limnothrix* (strain AC0243) producing a neurotoxin requires further investigation. 

Recent findings have identified that BMAA exposure causes a significant decrease in glucose concentrations recovered from the serum of treated animals [13]. When the stages in energy pathways are considered, decreased glucose levels would result in significant reduction in cellular ATP levels [51]. Subsequently, the reduction of glucose may impede brain development due to the brain’s high energy demand [16]. In striking similarity, Vero cell lines exposed to *Limnothrix* (strain AC0243) also exhibited a reduction in cellular ATP levels [1,2].

This reduction of ATP concentrations coupled with the pathologies exhibited by the mouse bioassays and the *B. marinus* larvae suggest a possible link between *Limnothrix* (strain AC0243) and BMAA. The mouse bioassays using *Limnothrix* (strain AC0243) extracts demonstrated weakness of the limbs and loss of coordination [2], symptoms typical of BMAA exposure [3,14]. Although behaviourally, the majority of *B. marinus* larvae exposed to live cultures of *Limnothrix* (strain AC0243) did not show any measurable signs of neuropathy, two animals were witnessed convulsing, also symptomatic of BMAA exposure [3,14]. Histological examinations showed brain damage in the treated larvae. Control animals were not impacted.

Differences in histopathologies between the mouse bioassays and the *B. marinus* larvae may be explained by the developmental stage of the two animal groups. Tests have shown areas such as the liver and developing brain of neonatal rats with limited blood brain barrier protection and greater protein synthesis are more susceptible to BMAA uptake [5,16]. Animals in early development such as at embryonic and larval stages where the notochord is forming or metamorphosing are reportedly more susceptible to toxicosis [52,53].

In contrast, adult mice have shown only limited uptake of BMAA in the brain, likely due to greater blood brain barrier protection, with the majority of uptake occurring in the renal and hepato-pancreatic regions [54]. Similarly, the mouse bioassays showed histological evidence of extensive liver damage without obvious brain injuries [2]. *Bufo marinus* larvae exhibited cellular damage in both regions, also with damage predominantly occurring in the hepato-pancreatic region. The uptake of BMAA has been shown to be greater in areas such as the hepato-pancreatic region where protein synthesis is greater [16].

The histopathologies seen in the gastrointestinal tract of the larvae were comparatively different to those reported in the mouse bioassays. The significant narrowing to complete closure of the GI tract of the treated larvae could also have been a secondary effect of neuropathy or notochord injury. The notochord critically contributes to the patterning of the development of the nervous system by secreting sonic hedgehog (shh) proteins, a primary factor regulating organogenesis [41,55,56]. Notochord abnormalities have been shown contribute to other pathologies such as foregut, sacral, and anorectal malformations [38,57]. It has also been implicated in the development of sympathoadrenal progenitors and heart formation [37].

Other possibilities explaining the differences in toxicosis include treatment preparation and the route of toxin uptake. Previous research has determined the toxin(s) to be water soluble, possibly further enhancing toxicosis in exposed larvae [1,2]. Aqueous solutions are readily absorbed cutaneously in anurans, providing an inlet for toxins, as well as maximising the potential for transdermal uptake [27,28,29,30,31,58]. Grazing habits of the larvae may have also enhanced toxicosis. Oral ingestion of whole cells has been shown to be significantly more toxic than orally ingested/injected cell extracts [42,59]. 

Toxic cyanobacterial blooms have been shown to cause adverse environmental impacts, eliciting mass mortalities in fauna [60,61,62,63,64,65]. Despite this, toxic cyanobacteria are seldom considered in terms of declining anuran populations [43]. The effects of the toxin(s) produced by *Limnothrix* (strain AC0243) has shown in this study, on short term exposure to 100,000 cells mL^−1^, to cause pernicious injuries to anuran larvae on an individual level, but further imply the likelihood of impacting local communities and populations. Furthermore, the ramifications of the toxicosis in larvae could also impact predator populations, as larvae in part contribute as a food source for animals higher in the food chain [66,67,68]. 

## 3. Experimental Section

### 3.1. Limnothrix (Strain AC0243) Culture Collection and Preparation

Single trichome isolates of the original material described in [1] were used in these experiments. The isolates were added to ASM-1 media made as described in [69] in sterile non-treated Sarstedt (Nümbrecht, Germany) 75 cm^2^ tissue culture flasks with ventilated lids. The inoculated flasks were placed in a climate controlled room set for 27 °C, under a 14/10 h diurnal regime at a light intensity of 10 µE m^2^ s^−1^ and subsequently left to grow into dense colonies. 

### 3.2. Larvae Collection and Test Preparation

Methods for the experiment were derived from [32,42]. *Bufo marinus* larvae at approximately Porter [70] development stage 33 were collected from a privately owned dam in Cawarral, Central Queensland. The dam had no known history of cyanobacterial blooms. The larvae were netted and transferred to a 10 L bucket containing dam water and transported back to Central Queensland University, Rockhampton. Additional water was collected in BMW Plastics (Dandenong, Victoria, Australia) 20 L plastic water containers and filtered using Whatmans (Kent, UK) GF/F glass filters in a Nalgene^®^ (Rochester, New York, NY, USA) vacuum filtration unit as prescribed by [18,32]. Prior to filtration, five random samples of the water were analysed using PYSER (Kent, UK) 1 mL/1 µL Sedgewick Rafter counting chambers, to ensure no toxic cyanobacteria were present in the water. The filtered dam water was used as the control water for the experiments to minimize any stress on the animals resulting from a change of water conditions. 

The collected larvae were placed in labeled 500 mL wide neck Bomex (Beijing, China) Erlenmeyer flasks (one larva per flask, seven replicates per treatment) in a climate controlled laboratory set at 28 °C, with a 12/12 h artificial diurnal cycle. The flasks were not aerated, to maintain the dissolved oxygen at approximately 40% saturation, mimicking the larvae’s natural environment. Each flask was randomly placed in rows. The larvae were fed pre-boiled lettuce and left to acclimatise for 48 h. After the acclimatisation period and every second day for seven days, two thirds of the flask constituents were carefully decanted and replaced with control water for the controls or test water consisting of 100,000 cells mL^−1^ of *Limnothrix* (strain AC0243) live cultures. This concentration of cells was chosen as it is the cell concentration considered by the World Health Organisation as the basis for Alert Level 2 in their monitoring management framework [7]. The correct concentration was achieved by diluting stock cultures with control water. The concentration was checked prior to water changes using a PYSER (Kent, UK) 1 mL/1 µL Sedgewick Rafter counting chamber. After each water change for the duration of the experiment, the flasks in both the control and treatment groups were randomly repositioned in rows and approximately 5 g of pre-boiled lettuce were added to each flask.

### 3.3. Water Quality Testing

The decanted flask constituents from section 3.2 were tested for dissolved oxygen (% saturation), pH and temperature (°C), using a TPS Australia (Springwood, New South Wales, Australia) WP-91 meter immediately after each water change. Ammonia concentrations were also measured using Salifert (Duiven, Holland) ammonia testing kits. Discarded test solution (10 mL) from each flask was transferred to test tubes with corresponding labels and analysed as per the manufacturer’s instructions. 

### 3.4. Euthanasia of Animals

On completion of the experiment or when animals were deemed moribund, each animal was anaesthetised by being placed in iced water, followed by submersion in a Sarstedt (Nümbrecht, Germany) 50 mL Centrifuge tube with 25 mL of 10% neutral buffered formalin for euthanization. The 10% neutral buffered formalin was pre-chilled to maintain anaesthetisation during euthanasia.

### 3.5. Histology

All histology was carried out by Histology Services, Bundaberg, Australia. The larvae were manually processed and embedded in paraffin wax. Transverse sections were processed at 4 µm thicknesses and haematoxylin and eosin (H&E) stained.

After wax mounting, transverse sections were processed at 4 µm thicknesses in ribbons of 4 to 5 sections with 50 µm between ribbons. A LKB (Vienna, Austria) Rotary-ONE microtome was used with Feather (Osaka, Japan) S35 disposable microtome blades for sectioning. The sections were placed on Livingstone International (Rosebery, New South Wales, Australia) premium quality glass slides and deparaffinised. The deparaffinised sections were then stained with Amber Scientific (Eugene, OR, USA) HH 2.5 L Harris’ haematoxylin and eosin stain.

Coverslips were mounted using Sigma Aldrich (Castle Hill, New South Wales, Australia) D.P.X mounting resin and left to dry. The sections were analysed using a Nikon (Tokyo, Japan) Eclipse E200 binocular microscope and photographed using a Nikon (Tokyo, Japan) Digital sight DS-L1 camera under 40×, 100×, and 400× magnifications as specified in each figure. 

## 4. Conclusions

Reports of *Limnothrix* (strain AC0243) derived poisoning events have not yet been documented as the toxicity of this strain has only recently been detected. *Limnothrix* secies are usually found growing in conjunction with *C. raciborskii* [51,52,53,54]. Consequently, this strain has been overlooked as a causal agent in poisoning events. 

Previous trials have established acute toxicity of *Limnothrix* (strain AC0243) in mammals [1,2].

The results of this experiment indicate that the toxin(s) produced by *Limnothrix* (strain AC0243) causes severe toxicosis to developing vertebrates with injuries particularly noted in the brain, notochord and pancreas. The damage caused by this strain showed strong similarities to BMAA intoxication and further investigation is recommended. The systemic toxicosis caused by the toxin(s) indicates that this strain has the potential to significantly impact upon aquatic populations. Research is also needed to evaluate the concentration dependent effects of the toxin(s) as well as explore the long-term consequences of exposure.

## References

[1] Bernard C., Froscio S., Campbell R., Monis P., Humpage A., Fabbro L. (2011). Novel toxic effects associated with a tropical *Limnothrix /Geitlerinema*-like cyanobacterium. Environ. Toxicol..

[2] Humpage A., Falconer I., Bernard C., Froscio S., Fabbro L. (2012). Toxicity of the cyanobacterium *Limnothrix* AC0243 to male balb/C mice. Water Res..

[3] Aráoz R., Molgó J., Marsac N.T.D. (2010). Neurotoxic cyanobacterial toxins. Toxicon.

[4] Carmichael W. (2001). Health effects of toxin-producing cyanobacteria: “The cyanoHABs”. Hum. Ecol. Risk Assess..

[5] Karlsson O., Lindquist N.G., Brittebo E.B., Roman E. (2009). Selective brain uptake and behavioral effects of the cyanobacterial toxin BMAA (β-*N*-methylamino-l-alanine) following neonatal administration to rodents. Toxicol. Sci..

[6] Watkins S., Reich A., Fleming L., Hammond R. (2008). Neurotoxic shellfish poisoning. Mar. Drugs.

[7] World Health Organisation (WHO) (1999). Toxic Cyanobacteria in Water: A Guide to Their Public Health Consequences, Monitoring and Management.

[8] Chiu A.S., Gehringer M.M., Braidy N., Guillemin G.J., Welch J.H., Neilan B.A. (2012). Excitotoxic potential of the cyanotoxin β-methyl-amino-l-alanine (BMAA) in primary human neurons. Toxicon.

[9] Okle O., Rath L., Galizia C.G., Dietrich D.R. (2013). The cyanobacterial neurotoxin beta-*N*-methylamino-l-alanine (BMAA) induces neuronal and behavioral changes in honeybees. Toxicol. Appl. Pharmacol..

[10] Brand L.E., Pablo J., Compton A., Hammerschlag N., Mash D.C. (2010). Cyanobacterial blooms and the occurrence of the neurotoxin, beta-*N*-methylamino-l-alanine (BMAA), in South Florida aquatic food webs. Harmful Algae.

[11] Purdie E.L., Samsudin S., Eddy F.B., Codd G.A. (2009). Effects of the cyanobacterial neurotoxin β-*N*-methylamino-l-alanine on the early-life stage development of zebrafish (*Danio rerio*). Aquat. Toxicol..

[12] De Munck E., Muñoz-Sáez E., Miguel B.G., Solas M.T., Ojeda I., Martínez A., Gil C., Arahuetes R.M. (2013). β-*N*-Methylamino-l-alanine causes neurological and pathological phenotypes mimicking Amyotrophic Lateral Sclerosis (ALS): The first step towards an experimental model for sporadic ALS. Environ. Toxicol. Pharmacol..

[13] Engskog M.K.R., Karlsson O., Haglöf J., Elmsjö A., Brittebo E., Arvidsson T., Pettersson C. (2013). The cyanobacterial amino acid β-*N*-methylamino-l-alanine perturbs the intermediary metabolism in neonatal rats. Toxicology.

[14] Karamyan V.T., Speth R.C. (2008). Animal models of BMAA neurotoxicity: A critical review. Life Sci..

[15] Karlsson O., Berg A.L., Lindström A.K., Hanrieder J., Arnerup G., Roman E., Bergquist J., Lindquist N.G., Brittebo E.B., Andersson M. (2012). Neonatal exposure to the cyanobacterial toxin BMAA induces changes in protein expression, and neurodegeneration in adult hippocampus. Toxicol. Sci..

[16] Karlsson O., Jiang L., Andersson M., Ilag L.L., Brittebo E.B. (2014). Protein association of the neurotoxin and non-protein amino acid BMAA (β-*N*-methylamino-l-alanine) in the liver and brain following neonatal administration in rats. Toxicol. Lett..

[17] Goto J.J., Koenig J.H., Ikeda K. (2012). The physiological effect of ingested β-*N*-methylamino-l-alanine on a glutamatergic synapse in an *in vivo* preparation. Comp. Biochem. Physiol. Part C.

[18] Leeuwangh P., Kappers F.I., Dekker M., Koerselman W. (1983). Toxicity of cyanobacteria in Dutch lakes and reservoirs. Aquat. Toxicol..

[19] Falconer I.R. (1989). Effects on human health of some toxic cyanobacteria (blue-green algae) in reservoirs, lakes and rivers. Toxic. Assess..

[20] Repavich W.M., Sonzogni W.C., Standridge J.H., Wedepohl R.E., Meisner L.F. (1990). Cyanobacteria (blue-green algae) in Wisconsin waters: Acute and chronic toxicity. Water Res..

[21] Carmichael W. (1992). Cyanobacteria secondary metabolites-the cyanotoxins. J. Appl. Bacteriol..

[22] Negri A., Jones G. (1995). Bioaccumulation of Paralytic Shellfish Poisoning (PSP) toxins from the cyanobacterium *Anabaena circinalis* by the freshwater mussel *Alathyria condola*. Toxicon.

[23] Negri A.P., Jones G.J., Hindmarsh M. (1995). Sheep mortality associated with paralytic shellfish poisons from the cyanobacterium *Anabaena circinalis*. Toxicon.

[24] Falconer I.R. (1999). An overview of problems caused by toxic blue–green algae (cyanobacteria) in drinking and recreational water. Environ. Toxicol..

[25] Ernst B., Hoeger S.J., O’Brien E., Dietrich D.R. (2006). Oral toxicity of the microcystin-containing cyanobacterium *Planktothrix rubescens* in European whitefish (*Coregonus lavaretus*). Aquat. Toxicol..

[26] Brand L.E., Campbell L., Bresnan E.K. (2012). The biology and ecology of a toxic genus. Harmful Algae.

[27] Uchiyama M., Konno N. (2006). Hormonal regulation of ion and water transport in anuran amphibians. Gen. Comp. Endocrinol..

[28] Azevedo R.A., Carvalho H.F., de Brito-Gitirana L. (2007). Hyaluronan in the epidermal and the dermal extracellular matrix: Its role in cutaneous hydric balance and integrity of anuran integument. Micron.

[29] Maejima S., Yamada T., Hamada T., Matsuda K., Uchiyama M. (2008). Effects of hypertonic stimuli and arginine vasotocin (AVT) on water absorption response in Japanese treefrog, *Hyla japonica*. Gen. Comp. Endocrinol..

[30] Suzuki M., Tanaka S. (2009). Molecular and cellular regulation of water homeostasis in anuran amphibians by aquaporins. Comp. Biochem. Physiol. Part A.

[31] Maejima S., Konno N., Matsuda K., Uchiyama M. (2010). Central angiotensin II stimulates cutaneous water intake behavior via an angiotensin II type-1 receptor pathway in the Japanese tree frog *Hyla japonica*. Horm. Behav..

[32] White S.H., Duivenvoorden L.J., Fabbro L.D., Eaglesham G.K. (2007). Mortality and toxin bioaccumulation in *Bufo marinus* following exposure to *Cylindrospermopsis raciborskii* Cell extracts and live cultures. Environ. Pollut..

[33] Junges C.M., Peltzer P.M., Lajmanovich R.C., Attademo A.M., Cabagna Zenklusen M.C., Basso A. (2012). Toxicity of the fungicide Trifloxystrobin on tadpoles and its effect on fish-tadpole interaction. Chemosphere.

[34] Gillespie G.R. (2002). Impacts of sediment loads, tadpole density, and food type on the growth and development of tadpoles of the spotted tree frog *Litoria spenceri*: An in-stream Experiment. Biol. Conserv..

[35] Lamberti G.A., Feminella J.W., Pringle C.M. (2007). Methods in Stream Ecology.

[36] Tyler M. (2009). Frogs and Toads as Experimental Animals.

[37] Stemple D., Solnica-Krezel L., Zwartkruis F., Neuhauss S.C., Schier A.F., Malicki J., Stainier D.Y., Abdelilah S., Rangini Z., Mountcastle-Shah E. (1996). Mutations affecting development of the notochord in zebrafish. Development.

[38] Qi B.Q., Beasley S.W., Frizelle F.A. (2003). Evidence that the notochord may be pivotal in the development of sacral and anorectal malformations. J. Pediatr. Surg..

[39] Platz F. (2006). Structural and experimental investigations of the functional anatomy and the turgor of the notochord in the larval tail of anuran tadpoles. Ann. Anat..

[40] Pagnon-Minot A., Malbouyres M., Haftek-Terreau Z., Kim H.R., Sasaki T., Thisse C., Thisse B., Ingham P.W., Ruggiero F., Le Guellec D. (2008). A novel factor in zebrafish notochord differentiation and muscle development. Dev. Biol..

[41] Ribes V., Balaskas N., Sasai N., Cruz C., Dessaud E., Cayuso J., Tozer S., Yang L.L., Novitch B., Marti E. (2010). Distinct sonic hedgehog signaling dynamics specify floor plate and ventral neuronal progenitors in the vertevrate neural tube. Genes Dev..

[42] Kinnear S.  (2006). Cylindrospermopsin in whole cell extracts and live cultures of *Cylindrospermopsis raciborskii*: Ecotoxicity, bioaccumulation and management. PhD thesis.

[43] Ziková A., Lorenz C., Lutz I., Pflugmacher S., Kloas W. (2013). Physiological responses of *Xenopus laevis* tadpoles exposed to cyanobacterial biomass containing microcystin-LR. Aquat. Toxicol..

[44] National Health and Medical Research Council (2004). Australian Code of Practice for the Care and Use of Animals for Scientific Purposes.

[45] Gao Y., Cornwell J.C., Stoecker D.K., Owens M.S. (2012). Effects of cyanobacterial-driven pH increases on sediment nutrient fluxes and coupled nitrification-denitrification in a shallow fresh water estuary. Biogeosciences.

[46] Marques S.M., Chaves S., Gonçalves F., Pereira R. (2013). Evaluation of growth, biochemical and bioaccumulation parameters in * Pelophylax perezi* tadpoles, following an *in-situ* acute exposure to three different effluent ponds from a uranium mine. Sci. Total Environ..

[47] ANZECC (2000). Australian and New Zealand Guidelines for Fresh and Marine Water Quality.

[48] Seigel R., Dismore A., Richter S. (2006). Using well water to increase hydroperiod as a management option for pond-breeding amphibians. Wildl. Soc. Bull..

[49] Markula A., Csurhes S., Hannan-Jones M. (2010). Economic Development and Inovation Department of Employment.

[50] Faassen E.J., Harkema L., Begeman L., Lurling M. (2012). First report of (Homo)Anatoxin-a and dog neurotoxicosis after ingestion of benthic cyanobacteria in the Netherlands. Toxicon.

[51] Ioudina M., Uemura E., Greenlee H.W. (2004). Glucose insufficiency alters neuronal viability and increases susceptibility to glutamate toxicity. Brain Res..

[52] Berry J.P., Gantar M., Gibbs P.D.L., Schmale M.C. (2007). The zebrafish (*Danio rerio*) embryo as a model system for identification and characterization of developmental toxins from marine and freshwater microalgae. Comp. Biochem. Physiol. Part C.

[53] Stewart I. (2010). Environmental risk factors for temporal lobe epilepsy—Is prenatal exposure to the marine algal neurotoxin domoic acid a potentially preventable cause?. Med. Hypotheses.

[54] Karlsson O., Berg C., Brittebo E.B., Lindquist N.G. (2009). Retention of the cyanobacterial neurotoxin β-*N*-Methylamino-l-Alanine in melanin and neuromelanin-containing cells—A possible link between Parkinson-dementia complex and pigmentary retinopathy. Pigment Cell Melanoma Res..

[55] Müller F., Albert S., Blader P., Fischer N., Hallonet M., Strähle U. (2000). Direct actin of the nodal-related signal cyclops in induction of sonic hedgehog in the ventral midline of the CNS. Development.

[56] Lek M., Dias J.M., Marklund U., Uhde C.W., Kurdija S., Lei Q., Sussel L., Rubenstein J.L., Matise M.P., Arnold H.H. (2010). A homeodomain feedback circuit underlies step-function interpretation of a Shh morphogen gradient during ventral neural patterning. Development.

[57] Qi B.Q., Beasley S.W. (1999). Relationship of the notochord to foregut development in the fetal rat model of esophageal atresia. J. Pediatr. Surg..

[58] McDairmid R., Altig R. (1999). Tadpoles: The Biology of Anuran Larvae.

[59] Kankaanpää H.T., Holliday J., Schröder H., Goddard T.J., von Fister R., Carmichael W.W. (2005). Cyanobacteria and prawn farming in Northern New South Wales, Australia—A case study on cyanobacteria diversity and hepatotoxin bioaccumulation. Toxicol. Appl. Pharmacol..

[60] Quiblier C., Susanna W., Isidora E.S., Mark H., Aurélie V., Jean-François H. (2013). A review of current knowledge on toxic benthic freshwater cyanobacteria—Ecology, toxin production and risk management. Water Res..

[61] Handeland K., Østensvik Ø. (2010). Microcystin poisoning in roe deer (*Capreolus capreolus*). Toxicon.

[62] Krienitz L., Ballot A., Kotut K., Wiegand C., Pütz S., Metcalf J.S., Codd G.A., Pflugmacher S. (2003). Contribution of hot spring cyanobacteria to the mysterious deaths of lesser flamingos at Lake Bogoria, Kenya. FEMS Microbiol. Ecol..

[63] Lopez Rodas V., Costas E. (1999). Preference of mice to consume *Microcystis aeruginosa* (toxin-producing cyanobacteria): A possible explanation for numerous fatalities of livestock and wildlife. Res. Vet. Sci..

[64] Soll M., Williams M. (1985). Mortality of white rhinoceros (*Ceratotherium simum*) Suspected to be associated with the blue-green alga (*Microcystis aeruginosa*). J. S. Afr. Vet. Assoc..

[65] Stewart I., Seawright A.A., Shaw G.R., Hudnell H. (2008). Advances in Medicine and Biology.

[66] Jara F.G., Perotti M.G. (2010). Risk of predation and behavioural response in three anuran species: Influence of tadpole size and predator type. Hydrobiologia.

[67] Kiesecker J., Chivers D.P., Marco A., Quilchano C., Anderson M.T., Blaustein A.R. (1999). Identification of a disturbance signal in larval red-legged frogs, *Rana aurora*. Anim. Behav..

[68] Mirza R.S., Ferrari M.C.O., Kiesecker J.M., Chivers D.P. (2006). Responses of american toad tadpoles to predation cues: Behavioural response thresholds, threat-sensitivity and acquired predation recognition. Behaviour.

[69] Gorham P., Lachlan J.M., Hammer U., Kim W. (1964). Isolation and culture of toxic strains of *Anabaena flos-aquae* (Lyngb) De Breb. Verh. Int. Ver. fuer Theor. und Angew. Limnol..

[70] Porter K. (1972). Herpetology.

